# The DWQ-EMR Embedded Tool to Enhance the Family Physician-Caregiver Connection: A Pilot Case Study

**DOI:** 10.3390/geriatrics6010029

**Published:** 2021-03-21

**Authors:** Kristina Marie Kokorelias, Einat Danieli, Sheila Dunn, Sid Feldman, David Patrick Ryan, Joel Sadavoy

**Affiliations:** 1 St. John’s Rehab Research Program, Sunnybrook Health Sciences Research Institute, Sunnybrook Health Sciences Centre, 285 Cummer Avenue, Room B105, Toronto, ON M2M 2G1, Canada; kristina.kokorelias@sunnybrook.ca; 2 Baycrest Health Sciences, 3560 Bathurst St, Toronto, ON M6A 2E1, Canada; EDanieli@baycrest.org (E.D.); sFeldman@baycrest.org (S.F.); 3 Women’s College Research Institute, Women’s College Hospital, 790 Bay St, Toronto, ON M5G 1N8, Canada; Sheila.Dunn@wchospital.ca; 4 Faculty of Medicine, University of Toronto, 263 McCaul St 3 Fl, Toronto, ON M5T 1W7, Canada; David.Ryan@sunnybrook.ca; 5 Department of Psychiatry, Sinai Health, 60 Murray St, Toronto, ON M5P 3C3, Canada

**Keywords:** Alzheimer’s disease, caregivers, dementia, primary care, family physicians

## Abstract

The number of family caregivers to individuals with dementia is increasing. Family physicians are often the first point of access to the health care system for individuals with dementia and their caregivers. Caregivers are at an increased risk of developing negative physical, cognitive and affective health problems themselves. Caregivers also describe having unmet needs to help them sustain care in the community. Family physicians are in a unique position to help support caregivers and individuals with dementia, but often struggle with keeping up with best practice dementia service knowledge. The Dementia Wellness Questionnaire was designed to serve as a starting point for discussions between caregivers and family physicians by empowering caregivers to communicate their needs and concerns and to enhance family physicians’ access to specific dementia support information. The DWQ aims to alert physicians of caregiver and patient needs. This pilot study aimed to explore the experiences of physicians and caregivers of people using the Questionnaire in two family medicine clinics in Ontario, Canada. Interviews with physicians and caregivers collected data on their experiences using the DWQ following a 10-month data gathering period. Data was analyzed using content analysis. Results indicated that family physicians may have an improved efficacy in managing dementia by having dementia care case specific guidelines integrated within electronic medical records. By having time-efficient access to tailored supports, family physicians can better address the needs of the caregiver–patient dyad and help support family caregivers in their caregiving role. Caregivers expressed that the Questionnaire helped them remember concerns to bring up with physicians, in order to receive help in a more efficient manner.

## 1. Introduction

Worldwide, the proportion of persons living with dementia is rapidly growing [1,2]. Due to the complex cognitive and physical changes that occur in persons living with dementia (PWD), individuals with the condition often rely on family caregivers (caregivers) to support them with their activities of daily living [3,4,5]. Caring for a PWD has significant, negative implications for caregivers’ health and well-being [6]. Seminal research has found that in comparison to caregivers of individuals with other chronic conditions, caregivers to PWD experience greater impacts on their employment, strain, mental and physical health and family dynamics [7]. Caregivers to individuals with PWD also report needs that are unmet by healthcare professionals compared to other family caregivers, that in turn may impact their physical and mental health [8,9]. Research also suggests that caregivers’ difficulty in sustaining their caregiving role can result in premature institutionalization and increased health-service utilization, by PWD and their caregivers [7,10,11]. Comprehensive primary health care supports for caregivers may delay institutionalization and reduce negative consequences of caregiving [12]. With the current shortage of specialists such as behavioral neurologists, geriatricians, and geriatric psychiatrists [13], improving caregivers’ access to existing health professional resources is of paramount importance.

In addition to diagnosing dementia and discussing disease prognosis and treatment plans, FPs are uniquely positioned to support caregivers, who are often not their patients [14], by advising on how to manage behavioral symptoms, and linking caregivers with appropriate health care services for themselves and PWD [12]. FPs are often the first point of access to the health care system for PWD and their family caregivers [12,15,16]. From a clinical perspective, numerous studies highlight the importance of the family member as a third person within geriatric medical encounters [17,18], as FPs often rely on caregivers for reliable information about the PWD, due to the memory challenges of their patients [19]. Overall, the examination of the caregiver–dementia care recipient dyad is often overlooked by FPs as the unit of dementia care and consequently, all treatment interventions must recognize the live-in family caregiver–patient dyad [20]. Caregivers have reported considerable communication problems during their interactions with physicians which limits their acquiring of knowledge and information relevant to their relative’s care [21,22]. Likewise, FPs struggle to communicate with caregivers to PWD [23,24] and may not be aware of caregivers’ increased stress and use of health service resources [25]. FPs report frustration with the role expectations of caregivers and their lack of capacity to support PWD [26,27] because of their lack of knowledge and time to learn about appropriate community health service resources (e.g., allied health, support groups) [28,29,30]. In situations where FPs know of community resources, they often feel unsure whether caregivers will be receptive to their input [28]. As such, caregivers to PWD perceive FPs as being unhelpful in making referrals to support resources [30,31]. There is a paucity of research about how to best empower caregivers to communicate their needs with FPs. There also appears to be a need for tools and processes to support FPs in addressing needs of caregivers and learning about and accessing community resources [28,31].

The Dementia Wellness Questionnaire (DWQ) was developed to enhance the capacity of FPs to clinically support both patients and caregivers of PWD. The DWQ is a self-administered electronic questionnaire designed to be completed on a privacy compliant tablet system by caregivers. This could occur in the FPs waiting room or on their home computer prior to their appointment. The questionnaire is structured to collect information, in an organized way, about issues related to the PWD and the caregiving situation to identify caregivers’ support needs, and risks to the well-being of caregivers or PWD. The questionnaire was developed after a vast literature review and focus group testing and expert review. Most importantly, the DWQ system is designed to synchronize with the FP’s electronic medical records (EMRs) system to provide FPs with real-time clinical data for both the PWD and their caregiver. The DWQ synchronizes with the patient’s EMR record and generates: (a) a summary of responses with high-risk areas highlighted in red (e.g., risk of falls); (b) best practice suggestions derived from evidence-based guidelines and practices; (c) links to suggested, specific tools relevant to the issues raised by the caregiver on the DWQ; (d) direct links to the referral forms for relevant services/referrals to community-based organizations and services for the caregiver and PWD and; (e) a custom-made printable handout for the caregiver, outlining all recommended services with descriptions and contact information. Literature on EMR use in primary care suggests that FPs prefer the organization, and readability of data within the EMR system as it reduces physicians’ need to recall information from memory and contributes to their efficiency in patient communication [32,33]. However, the clinical impact of using customizable functionalities that are designed to better support the needs of dementia patients and their caregivers is still to be determined.

Health service research priorities include the use of patient and caregivers’ perceptions to improve primary care to better meet patients’ and caregivers’ evolving needs and demand for service excellence [34,35]. As such, the key aim of this pilot case study was to test the utility and content relevance of the DWQ for both caregivers and FPs.

## 2. Materials and Methods

### 2.1. Design

A multi-method design was employed for this pilot case study [36,37]. Case study methodology was chosen to gain a concrete and contextual understanding about the specific use of the DWQ in two settings [38]. REB approval was received from Women’s College Hospital and Sinai Health Systems.

### 2.2. Participants and Recruitment

Seven FPs who treat patients with any type of dementia were recruited from two sites in Ontario, Canada (3 from site A and 4 from site B). Site A was an interprofessional academic Family Health Team located in a large urban hospital and site B was a community-based family health care clinic. FP participants identified and invited 1–2 caregivers of PWD to participate. Participants had to have had at least two visits within a 10-month period to be included. FPs were encouraged to recruit caregivers of different genders, ethnic backgrounds and socio-economic statuses. A member of the research team (K.M.K.) contacted interested caregivers to ensure that they met inclusion criteria, reviewed the letter of information and obtained written consent. Caregivers had to be English speaking, self-identify as a primary caregiver and self-identify as having basic computer literacy skills. Participants were excluded if they reported any discomfort with using computer programs and other applications associated with computers and tablets. Eleven caregivers participated in the study (4 from site A and 7 from site B).

### 2.3. Procedure

FPs attended a one-on-one 15-min training session on the EMR system with a trained research team member (K.M.K.). Following the training session, they were asked to use the information generated from the DWQ (linked through the EMR system) in their visits with PWD and/or family caregivers. FPs were shown how to access this information, how the information was populated, and provided contact information for the research team in the case of a challenge. Concurrently, family caregivers were first interviewed by a member of the research team about their expectations of the DWQ and any concerns they had and asked to complete the DWQ prior to each scheduled visit with the FP. At the time of the appointment booking or arrival at the clinic, the clinic receptionist provided instructions on completing the DWQ at home or clinic waiting room. Caregivers were instructed on how to access the DWQ on their computer or on the tablet in the FPs’ waiting area and an electronic link to the DWQ was sent to each participant’s email address. Assistance from clinic or research staff was available as needed. After completing the questionnaire, the PWD and caregiver met with the FP for their scheduled appointment. The caregiver’s responses to the DWQ synchronized with the patient’s EMR and the FP could immediately access the generated summary of problems/risks, guidelines, tools, and resources. Only the physician had access to the DWQ data. The research team did not have access to identifiable personal health information.

### 2.4. Data Collection

Demographic data for the caregiver was obtained using a paper-based demographic questionnaire prior to the first caregiver interview. Quantitative satisfaction data was gathered using a modified Primary Care Satisfaction Scale for Women’s Health, which has been evaluated in studies to evaluate the quality of primary care delivery [39]. Telephone interviews were used in order to increase participant confidentiality and privacy, and decrease cost and travel [40].

Qualitative data was derived from interviews of caregiver participants and FPs to explore perceptions of the DWQ. K.M.K. conducted all interviews and an interview guideline was designed to organize the interviews. Pre-intervention semi-structured interviews were conducted to explore caregivers’ expectations, concerns, and experiences communicating with FPs, as well as the anticipated benefits regarding the DWQ. After a trial period of 10 months, all FPs and caregivers participated in a post-intervention semi-structured interview regarding their experience with the DWQ tool and the usefulness of the summary output in guiding the provision of dementia care. After the interviews, caregiver participants completed a questionnaire about their experience with the DWQ. At the end of each participant interview, the interviewer provided the participant with a verbal summary of the interview to ensure that interpretation was correct. None of the participants disputed the interviewer’s summary.

### 2.5. Data Analysis

All interviews were professionally transcribed, checked for accuracy, de-identified of any personal information to ensure participant confidentiality and imported to NVivo software [41]. Transcripts were then analyzed using conventional content analysis guided by Hsieh and Shannon [42]. Conventional content analysis was selected as it does not include a preconceived notion of categories to fit participants’ responses in any ideas or theories but is rather derived from unique answers. First, all members of the research team independently reviewed the transcripts. Then K.M.K. coded the transcripts. To ensure intercoder reliability, the initial codes were consolidated into a codebook through an iterative process, which was then reviewed with the research team and approved for use to re-code the transcripts and search for themes within the data. Detailed data was reviewed by the research team. K.M.K. made notes of concepts and thoughts of the data. The research team also attended meetings to review and refine the re-coded data until a consensus on final themes was established. Questionnaire data was presented numerically (see tables below) and discussed amongst the team.

### 2.6. Rigor

The research team consisted of a qualitative researcher, an occupational therapist, a psychologist and three physicians who have experience working in the field of geriatrics. All team members have research and clinical expertise working directly with caregivers. Credibility and quality of the research process was ensured through strategies related to qualitative methodology. For instance, an audit trail containing meeting notes, analysis discussions, and research decisions was continuously maintained to ensure reliability of the research process [43]. The assumptions and reflections of all authors were discussed verbally during team meetings. These assumptions were also included in the audit trail. Reflexive notes were also taken after each interview so as to refine probes for subsequent interviews and reflect on the overall interview process [43]. To maximize the credibility of the findings, all authors reviewed data to contribute to the interpretation of the results [44].

## 3. Results

Eleven caregivers (4 from site A and 7 from site B) and 7 FPs (3 from site A and 4 from site B) participated in this study. The majority of caregiving participants were female (n = 8) and Caucasian (n = 9). Almost all of the caregiver participants were not the patient of the FPs. All but one FP participant were family doctors and not specialized geriatricians. Characteristics of caregiver participants are presented in Table 1 and FP participants in Table 2. Overall, caregivers and providers were supportive of the DWQ. Their comments are combined in the sections below and, in situations where their perspectives differed, these differences are described.

### 3.1. Caregiver Pre-Intervention Interviews

Caregivers hoped that using the DWQ would enhance their communication with FPs by highlighting their own key concerns, which they could then relay to the FP. Caregivers said that they often forgot their own concerns during visits, as the FP is normally focused on discussing the care plan of the care recipient. As one caregiver stated:

*How do I remember it all? I’m hoping the tool will help me remember it all so that I even know what my biggest issues are and then allow me to remember to tell the doctor about them and not just the issues of my mom (sic)*.

(Caregiver 3, Site A)

Caregivers expressed little concern about using the DWQ on a tablet, and only reported concerns about remembering to fill out the questionnaire before attending the appointments with FPs.

### 3.2. Caregiver Experiences Post-Interventions

All caregivers completed the DWQ on a tablet in the waiting room. Reasons for this preference ranged from liking having something to do in the waiting room to often forgetting to complete the survey at home.

Participants were very comfortable with completing the questionnaire and found it easy to access and navigate. One caregiver indicated that they would have liked the ability to pause when taking the survey and continue at a later time (Caregiver 4, Site A). Ten of the eleven caregivers shared that they gained comfort in knowing that they could ask the receptionists for help with any technical issues that they might encounter. One participant said that they would have preferred having a larger screen on which to complete the survey and more time to properly answer everything (Caregiver 7, Site B).

All of the caregivers felt that the DWQ prompts effectively captured their concerns. Nine of 11 participants said that the content of the survey was relevant to their situations. Two participants said that the questions about wandering were premature to their current experience. All but two caregivers deemed the questionnaire helpful with regard to visiting their family physicians. These two caregivers suggested that future iterations of the DWQ should include questions regarding caregiver needs for community services support. Again, all but two caregivers said that they would continue to use the questionnaire tool. Two said that they would not because they did not notice a big change after completing the survey.

### 3.3. Caregiver Satisfaction with Physician Pre/Post-Intervention

Caregivers expressed a great increase in trust in the physician following the use of the DWQ. Modest changes were noted in the caregivers’ perceptions about the FPs’ ability to clearly communicate and the amount of time they had to talk to the physician. No changes were noted in the FPs’ ability to answer questions in a sensitive and caring way, the chance of caregivers to ask all of their questions. Caregivers also perceived a great increase in the health professional’s willingness to explain different options for their care. Results of the pre-intervention and post-intervention questionnaires rating caregivers’ satisfaction with their visits are presented in Table 3.

### 3.4. Family Physician Post-Intervention Experiences

Family physicians talked about facilitators of DWQ-EMR implementation and discussed their perceptions of current and expected benefits of the system. Two physicians from Site A and three physicians from Site B spoke of the availability of a researcher “champion” (FP 2, Site A) being key to their uptake of the system. The champion provided support, helped solve problems, and was perceived as facilitating and maintaining enthusiasm for the transition to using the system.

All physicians reported that DWQ integration within the EMR system was efficient once they had learned the system. Some physicians (n = 4) described a challenge with first learning how to view results, but consulted with the champion at their organization. All physicians reported that making suggestions to caregivers for community services were much quicker using DWQ.

There were mixed reviews on the benefit of using the DWQ-EMR system for those FPs who were not the care physician for the caregiver, although four FPs reported that they were glad they could provide the caregiver with resources while also sending the information specific to the caregiver to the caregiver’s physician.

*“I like the premise of the tool, but if I am not the caregiver’s GP I don’t know why I am collecting the data. If I was, I’d see this being very useful. So all I did is print a form.”*

(FP 1, Site B)

All but one physician commented on feeling that focusing on caregivers’ health, even if they were not their primary care physician, was a necessity and that the DWQ could help them do so without creating “much extra work” (FP 2, Site A).

All of the family physicians emphasized that the DWQ needs to be adaptable to add new modules or functions over time. Several participants wished to add their own questions.

Physicians were surprised at how well caregivers used the tool. A few physicians had worried about the caregivers’ perceptions of the new technology. However, they observed that their patients’ reactions ranged from neutral to positive. Some caregivers occasionally even encouraged the physician to consult the DWQ tool and believed that the benefits of the DWQ would increase over time, with more routine use.

*“Actually I didn’t think they’d want to fill it out, but there were cases at the beginning I’d forget about it because not everyone I see is a dementia caregiver and the caregiver would pause and ask me if I saw the results from it.”*

(FP 4, Site B)

## 4. Discussion

This multi-method pilot case study explored implementation of an electronic tool for assessing and supporting the concerns and needs of caregivers for persons with dementia from the perspective of the caregivers and the dementia patients’ family physicians. Our findings suggest that the DWQ was developed to enhance dementia care in primary care by efficiently integrating the concerns of caregivers within the physician’s existing EMR system to enhance its usability and utility. We found that caregivers had no difficulty in using it and FPs believed that using the DWQ in practice helped them save time and allowed them to provide tailored resources to caregivers of persons with dementia, that may help them sustain their caregiving roles. Caregivers preferred to complete the forms in physicians’ offices and believed that the DWQ helped them remember concerns to bring up with physicians. Our findings also highlight FPs believe they should provide support to caregivers even if they are not their patients. Caregivers and family physicians noted that the physicians did not always access the DWQ results and sometimes needed prompting by the caregiver to do so. With continued use of the DWQ over time, physicians may be able to routinize the utilization of the output of the tool into discussions with family caregivers.

The lack of integration of care for both the caregiver and the PWD is recognized as a gap in the dementia caregiving system. The results of this study support capacity building in primary care to provide support for caregivers in the management of dementia. Sadavoy and Wesson [20] have advocated that the dyad of the PWD and caregiver be addressed as a single unit of care given the limited efficacy of medical interventions and reliance on the caregiver as the most critical instrument of care and intervention for dementia care at home. A few family physicians expressed that they did not feel it was their role to provide recommendation to a caregiver who is not their patient, and that such recommendations should be sent to the caregiver’s family physician instead. This is understandable given that FPs are both financially and possibly legally constrained in caring for caregivers who are not their patients. The current billing model in many jurisdictions does not provide incentives to physicians to provide integrated interventions for caregivers and the PWD. This is a potential structural problem in the caregiving system of the PWD. FPs recognized the inseparability of the caregiver and PWD. In support of integrated care, all but one physician believed that focusing on caregivers’ health, even if they were not their primary care physician, was becoming a necessity and that the DWQ could help them do so without creating “much extra work” (FP 2, Site 1).

Our findings suggest that when FPs used the DWQ, caregivers perceived that they received more information about resources and services from the doctor, suggesting that the tool may facilitate targeted discussion with the FP of caregiver needs and dementia management issues at home and further lead to practical and relevant support. It may also further enhance physician knowledge on dementia management including accessible community supports.

We have shown that supports for dementia care can be administered through EMRs in primary care settings, including caregiver-focused interventions, even in instances where the FP is not the caregiver’s health care provider. Although both caregivers and physicians found the DWQ easy to use, implementation required some instruction from the research team for its initial use. Clinic staff facilitators had to send electronic links for the DWQ to caregivers with an upcoming appointment otherwise coaching for caregivers using the tool on a tablet in the waiting room would be crucial to integration into the clinic workflow.

While caregivers had the option of filling out the DWQ at home, most filled out the survey in physicians’ offices. Barriers to general EMR use need to be considered, which may include the effect of the DWQ on EMR workflow, the need for on-going technological support, and wider-spread caregiver access to the DWQ from home. Future research would benefit from exploring other technological options for implementing the DWQ into practice, such as a mobile phone application highlighting caregiver support options. Additionally, in regard to the implementation and sustainability of incorporating the DWQ into practice, having staff facilitators to enable uptake of a DWQ into practice will be crucial. There is also the important need to maintain links to current resources for support, which may also be an issue. Best ways to support the sustainability and implementation of the DWQ requires further study.

### Limitations

This pilot study sample was a relatively homogenous convenience sample of Caucasian participants who had completed postsecondary education. Language, education, computer literacy and culture limit generalizability. Our findings may not reflect the experiences of the larger caregiving population or the caregiving experiences of individuals of different ethnicities. This study was only conducted at two sites in a large urban center in Toronto, Ontario and thus, may not fully reflect the large variety in practices across Canada, or elsewhere. This small pilot study lacked the power to see the impact of the tool on differential diagnosis, detection of risks and delirium as well as behavior management due to the small sample. The analysis provided is exploratory and preliminary. Moreover, future research on the effectiveness of the DWQ and the integration of it into routine practice might consider expanding the number of participants by recruiting from a larger geographical location (e.g., rural vs. urban), more diverse ethnicity group (e.g., culturally and linguistically diverse groups), and different family caregivers (e.g., spouses vs. adult children vs. paid caregivers) to determine if perceptions remain the same to what we have reported. Further work could also explore adapting the DWQ to facilitate PWD themselves to input their own symptoms and concerns to physicians.

## 5. Conclusions

New models of dementia care need to be developed and tested as the number of family members caring for people with dementia rise. The rise of EMR adoption in primary health care suggests that the DWQ may be valuable in assisting FPs in providing care for PWD and their family caregivers. In order to transform dementia care in the primary care sector, tools which aid family physicians in providing caregivers with useful information (such as the Dementia Wellness Questionnaire) should be brought to life and integrated within physicians’ everyday practices. This project provided us with insight on how we can modify and evaluate the tool in order to prepare it for dissemination to the wider primary care population. The DWQ were found to help FPs in getting the right information at the right time for the right patient. They will also augment communication between FPs and caregivers of PWD and, by extension, with PWD themselves. The integration of these two tools into practice can help contribute to goals of providing excellent care for residents who are aging at home with dementia, as these tools have been found to facilitate more efficient and direct access to dementia care guidelines. This has the potential to improve care for individuals with dementia living in the community and their caregivers by empowering caregivers to voice concerns to family physicians and for family physicians to stay up-to-date with the latest community resources.

## Figures and Tables

**Table 1:** Caregiver participant demographics.

Characteristics	Data Measure	Total Sample N = 11	Female N = 8	Male N = 3
Age	Mean (μ)	75.09	75	75.33
	Range	72 to 79	72 to 79	72 to 79
Ethnicity	Caucasian	9 (81.8%)	7 (87.5%)	2 (66.66%)
	Other	2 (18.1%)	1 (12.5%)	1 (33.33%)
Marital Status	Married/Common Law	11 (100%)	8 (100%)	3 (100%)
	Single	0	0	0
Completed postsecondary education	Yes	7 (63.63%)	4 (50%)	3 (100%)
Classification of care recipient’s dementia	Mild	4 (36.36%)	3 (37.5%)	1 (33%)
	Moderate	5 (45.45%)	3 (37.5%)	2 (67%)
	Severe	2 (18.18%)	2 (25%)	0
Provision of care *, total years	2–5 years	3 (27%)	0	3 (100%)
Provision of care *, hours per week	<6 h	0	0	0
	7–10 h	3 (27%)	3 (37.5%)	0
	11–15 h	1 (9%)	1 (12.5%)	0
	16–20 h	3 (27%)	3 (37.5%)	0
	25–35 h	1 (9%)	1 (12.5%)	0
	>36 h	0	0	0
Same FP as the PWD	Yes	1 (9%)	1 (12.5%)	0
	No	10 (91%)	7 (87.5%)	3 (100%)

* Median (interquartile range).

**Table 2:** Family physician participant demographics.

Characteristics	Data Measure	Total Sample N = 7	Female N = 4	Male N = 3
Age	Mean (μ) Range Standard Deviation	49.5 23	44.5 15	57 9
Residency and Fellowship Medical Training	Family Medicine Geriatrics	6 1	4 0	2 1
Additional Qualifications	MSc	2	2	0

**Table 3:** Satisfaction scale results regarding family physician pre- and post-intervention.

	Pre-Intervention			Post-Intervention		
Question Items	Mean Response *	Range *	Standard Deviation	Mean Response *	Range *	Standard Deviation
The amount of time I had to talk with the health professional…	3.25	2–5	1.09	3.5	2–5	1.02
The PWD’s health professional’s ability to answer questions in a sensitive and caring way…	3.25	2–5	1.09	3.25	3–5	1.09
The PWD’s health professional’s ability to explain things clearly…	3.25	2–5	1.09	3.5	2.5–5	1
The PWD’s health professional’s ability to help me feel comfortable talking about my concerns…	3.5	2–5	1.12	3.5	2–5	1.12
The chance to ask all of my questions…	3.25	2–5	1.09	3.25	2–5	1.09
The PWD’s health professional’s ability to take what I say seriously…	2.5	0–5	1.8	2.5	0–5	1.8
The PWD’s health professional’s willingness to explain different options for my care…	3.5	3–4	0.5	4	3.5–4	0.5
The PWD’s health professional’s interest in how my life affects my health…	1.5	0–3	1.5	1.5	0–3	1.5
The health professional’s knowledge of dementia-related issues…	4.25	2–5	1.30	4.5	2–5	1.00
The health professional’s interest in my mental and emotional health…	2	0–5	2.12	3	1–5	2.12
Help with finding information resources in dementia and/or caregiver care…	2	0–5	2.12	3	1–5	2.12
How well the health professionals explain the results of tests or procedure…	2	0–5	2.12	2	0–5	2.12
My overall trust in the health professional here…	2.25	0–5	0.43	4	3–5	0.5

* 1 signifies “Not at all satisfied”, 5 signifies “Extremely satisfied”.

## Data Availability

Not applicable.
